# Experiences of general practice teams and their patients with clinical research—a mixed-methods process evaluation of the Bavarian Research Practice Network (BayFoNet)

**DOI:** 10.1186/s12875-025-02744-x

**Published:** 2025-02-28

**Authors:** Linda Sanftenberg, Anna-Lena Schnaidt, Stefanie Eck, Antonius Schneider, Eva Bucher, Peter Konstantin Kurotschka, Ildikó Gágyor, Merle Klanke, Stefanie Stark, Thomas Kühlein, Fabian Walter, Marco Roos, Tobias Dreischulte, Jochen Gensichen, for the BayFoNet study group, Andrea Baumgärtel, Tobias Dreischulte, Stefanie Eck, Kathrin Lasher, Maike Ermster, Ildikó Gágyor, Jochen Gensichen, Alexander Hapfelmeier, Susann Hueber, Merle Klanke, Christian Kretzschmann, Thomas Kühlein, Peter Konstantin Kurotschka, Klaus Linde, Klara Lorenz, Marco Roos, Linda Sanftenberg, Antonius Schneider, Stefanie Stark, Til Uebel, Clara Teusen, Fabian Walter

**Affiliations:** 1 Institute of General Practice and Family Medicine, LMU University Hospital, LMU Munich, Nußbaumstr. 5, 80336 Munich, Germany; 2 Institute of General Practice and Health Services Research, Department Clinical Medicine, TUM School of Medicine and Health, Technical University of Munich (TUM), Munich, Germany; 3 Department of General Practice, University Hospital Würzburg, Würzburg, Germany; 4 Institute of General Practice, Friedrich-Alexander-University of Erlangen-Nuremberg, Erlangen, Germany; 5 General Practice, Medical Faculty, University of Augsburg, Augsburg, Germany

**Keywords:** General practice, Primary health care, Participatory research, Practice based research network, Implementation science

## Abstract

**Background:**

Practice-based research networks (PBRNs) have been implemented to support clinical research in German general practice since 2020. General practitioners (GPs) are often critical concerning the feasibility of clinical trials. Among others, high workload, lack of resources in GP teams and little acceptance of the trial requirements by the patients are assumed barriers. Therefore, the aim of this study was to evaluate the perspectives of the GP teams and their patients on the set up of BayFoNet during the implementation of the two pilot cluster-randomized trials to improve this collaboration on a sustainable basis.

**Methods:**

GPs and medical assistants (MAs) were interviewed using semi-structured interviews based on the Consolidated Framework for Implementation Research. Implementation research and verbatim transcripts were analyzed using qualitative content analysis. Patient attitudes were evaluated quantitatively with questionnaires based on the theoretical domains framework using descriptive statistics.

**Results:**

A total of 15 GPs and 15 MAs were interviewed, and 109 complete patient questionnaires were returned. Main facilitators for GPs’ active participation in clinical research were networking as well as active participation of GP teams at different levels of the research process. Increased awareness concerning PBRNs might promote a lively network. From the GPs’ perspective, lack of motivation among MAs and patients was a perceived barrier to support clinical research in general practice.

MAs emphasized their own increase in knowledge and competence as well as the importance of clinical research for improved patient care. In contrast to the GPs, most MAs were not aware of BayFoNet as a network structure. The surveyed patients rated their own capabilities and opportunities to actively participate in the pilot studies as very good. Prior to the implementations of the interventions, some patients experienced some difficulty in defining clear goals for their own participation.

**Discussion:**

Increased awareness concerning PBRNs might promote a lively network. Target-group specific dissemination strategies as well as opportunities for GP teams and their patients to participate in clinical research should be elaborated. This might increase the feasibility of clinical trials and the motivation of all participants to conduct clinical trials in general practice.

**Trial registration:**

Pilot cluster-randomized trial 1 (MicUTI) was prospectively registered on December 19, 2022 at www.clinicaltrials.gov (NCT05667207); Pilot cluster-randomized trial 2 (IMONEDA) was prospectively registered on April 22, 2022 at www.bfarm.de (DRKS00028805).

**Supplementary Information:**

The online version contains supplementary material available at 10.1186/s12875-025-02744-x.

## Introduction

Clinical research is mainly conducted in inpatient care, dealing with highly specialized issues. The results of these studies are often difficult to transfer to general practice [1, 2]. Therefore, scientific publications often provide no significant benefits for daily patient care in general practice [3]. However, GPs have to treat a wide range of conditions, see large numbers of patients with a huge variety of risk factors and medical conditions are managed over extended periods of time [4–6]. These circumstances offer a great opportunity and potentials to conduct clinical research in general practice.

In contrast to clinical research in inpatient care, general practices in Germany have no extra resources to develop or conduct clinical research, as they are independent market-oriented private businesses [7]. To support daily patient care, medical assistants (MAs) take on a variety of organizational tasks in German general practice, like ccoordinating patients’ appointments, reviewing and providing patient records and filing, preparation of invoices, sending letters, e-mails and SMS (for example patient reminders) or storage of medicines [8]. Due to the high workload in general practice, a lot of medical tasks are also delegated to them, e.g., performing laboratory tests and analyzing test results, preparing surgical treatment procedures, treating wounds, removing sutures, performing blood draws, swabs and injections, carrying out hygiene measures, recognizing infectious diseases and implementing protective measures [9].

The conduction of clinical trials includes many of those activities, such as the identification of eligible study participants, the distribution of study material, blood collection or swabbing, as well as the regular reminder of participating patients. As these activities do not require a medical qualification and are already part of the MAs work, MAs might play a major role in the successful implementation of clinical research in general practices [10]. The vocational qualification to work as an MA in Germany is achieved through three years of vocational education and in-company training [11].

However, a lack of opportunities and capabilities among MAs [12, 13] as well as missing motivation of patients to support clinical research in general practice are assumed barriers for successful implementation of clinical research in general practice [14, 15].

To establish clinical research in general practice on a sustainable basis, collaboration between general practice teams and universities is essential [1]. While countries such as the Netherlands, England, USA or Scotland [16–19] already have well-established practice-based research networks (PBRNs), the implementation of PBRNs in Germany is still in progress. Amongst six regional PBRNs in Germany, the German Federal Ministry of Education and Research (BMBF) is also funding a Bavarian Research Practice Network (BayFoNet) [20, 21] among five regional network centers (RNCs). Each RNC is responsible for practice recruitment. Practices that had already participated in other primary care research projects or were already part of a pre-existing teaching network have been invited to BayFoNet. Invitation to participate in a project had comprised an additional invitation to participate in BayFoNet. To be accredited in BayFoNet, all health care workers of GP practices had to obtain at least a basic qualification to conduct clinical studies. These training courses were organized by the coordination office of BayFoNet, were provided as web-based trainings and covered a clinically relevant topic in general practice as well as a topic relevant for clinical research in primary care. Study specific trainings, including training equipment and study material, were offered by each RNC face-to-face in the participating GP practices. BayFoNet offers regular meetings with GP teams to present upcoming clinical trials as well as the results of studies, that had been already finished. These meetings are organized every six months by another RNC and give the opportunity to network with the academic departments. To integrate the perspective of interested patient representatives and the general public, regular citizen-science meetings are organized by every RNC.

During the initial five-year funding period, two pilot cluster randomized controlled trials (RCTs) were conducted. As bad experiences with previous clinical trials can permanently destroy the GPs` motivation for further support of clinical research [22], it is of the utmost importance to understand the mechanisms of action for a successful implementation of clinical trials in BayFoNet [20].

Pilot cluster-randomized trial 1 evaluates the feasibility of a novel point-of-care test management to reduce antibiotic use in women’s uncomplicated urinary tract infections (MicUTI [23]). The MAs had to approach all adult women presenting with symptoms suggestive of acute uncomplicated urinary tract infection to identify trial participants. All approached patients were listed in paper-based anonymous patient pre-screening logs at each study site. MAs of the interventional arm performed phase-contrast microscopy after sufficient training to detect bacteria in a urine sample. Training was delivered face-to-face prior to patient recruitment over a three-hour session (baseline session), as well as three months after the start of patient recruitment (refresher session), following the principles of competency-based medical education.

Pilot cluster-randomized trial 2 investigates the effectiveness of an online education program for asthma patients in general practice with regard to asthma knowledge as primary outcome” (IMONEDA [24]). MAs were encouraged to identify eligible patients with a confirmed diagnosis of bronchial asthma, who had not yet attended an asthma education program or have not attended such program within the past ten years. After informed consent, they handed out the study material. Again, the approached patients were listed in paper-based anonymous patient pre-screening logs at each study site.

For an effective and sustainable collaboration between GP teams and academia, expectations and experiences of GP teams and their patients with an active participation have to be taken into account. The evaluated expectations of the GPs invited to participate in BayFoNet, before any clinical research have been conducted, referred mainly on their motivation to be part of BayFoNet. GPs aimed to contribute to evidence strength and quality in general practice, develop professionally and to get their GP team trained, as well as to network with academia and other GP colleagues. Reported barriers for an active participation were bad experiences with previous clinical studies as well as lack of available resources [22].

Therefore, the aim of this study was to evaluate the perspectives of the GP teams and their patients on the set up of BayFoNet during the implementation of the two pilot cluster-randomized trials to improve this collaboration on a sustainable basis.

## Material and methods

### Study design

We have designed a longitudinal mixed-methods process evaluation [20].

To identify barriers and facilitators from the perspectives of the GP teams following a theory-based approach, we referred to the consolidated framework for implementation research (CFIR).

The CFIR provides a conceptual model of implementation drivers across five domains, namely intervention characteristics, inner setting, outer setting, characteristics of the individual and process [25, 26].

In order to elicit barriers and facilitators from the perspectives of patients, we have drawn on the theoretical domains framework (TDF). This framework assumes that three key drivers (motivation, opportunity and capability) determine individual behavior (such as participation in research) [27]. To observe possible intervention-related changes among these key drivers for behavior change, the patients were surveyed before as well as during the interventions of the respective cluster-randomized pilot studies were carried out.

### Exploring the perspective of the GP teams

#### Recruitment

The GP teams were already members of BayFoNet and were actively involved in the pilot cluster-randomized trial 1 (MicUTI) or in the pilot cluster-randomized trial 2 (IMONEDA). We have invited the health care workers of all GP practices that have supported one of both pilot cluster-randomized trials for a qualitative semi-structured interview. As a compensation for their expenses, we have offered each interview participant €100.

#### Data collection

Interviews were conducted via telephone, video call or in person, according to the preferences of the study participants. No one else was present during these interviews besides the study participant and the researcher. GPs and MAs were interviewed individually.

The interviewing researcher was a female medical student (ALS) who conducted the qualitative study interviews in a self-reflective, neutral manner [28]. At the beginning of each interview, the interviewer introduced herself as a medical student and explained that the results of the interviews were to be used for her medical doctoral thesis. None of the interviews had to be repeated.

The transcripts were not returned to the participants for comments or correction. There was no feedback of the participants concerning the results or findings. Interviews were digitally recorded, transcribed verbatim, and pseudonymized thereafter. The interviews were conducted in parallel to the recruitment of interview partners. When further interviews added no additional themes and no further variance within themes, the data was assumed to be saturated and interviews were stopped.

The interview guide for the GPs referred to the motivation for an active participation in clinical research. In addition, we wanted to know whether the support provided by BayFoNet as an infrastructure was perceived as sufficient or whether there were any wishes for improvement. It was also asked how clinical trials have to be designed for a successful integration in general practice. In addition, we asked for perceived barriers in conducting RCTs in general practice (online Supplemental file 1). The interview guide for the MAs was part of a larger survey evaluating satisfaction with the study-specific training sessions and self-assessed competence growth. Therefore, we focused on two main aspects: the motivation to participate in clinical research in general, as well as the motivation to be a part of the PBRN BayFoNet (online Supplemental file 2).

#### Data analysis

Data was analyzed by means of structured content analysis according to Kuckartz [29], whereby deductive and inductive categories were formed. The five domains of the CFIR framework served as initial deductive codes. Text passages were coded supported by MAXQDA22, ordered, further substantiated in terms of content and systematized. By repeatedly reading the entire data set and applying the reduction steps according to Mayring [30], additional inductive codes were formed. The reporting to the qualitative analysis followed the consolidated criteria for reporting qualitative research (COREQ) (online Supplemental file 3) [31].

### Exploring the perspective of patients

#### Recruitment

We conducted a parallel survey, where data were collected simultaneously to the implementation of both pilot RCTs. We invited patients of both pilot RCTs at two different time points: before and during the intervention of the respective trial. The paper-based patient questionnaires were handed out by the GP team together with the respective study material of both trials.

Before the interventions started, questionnaires evaluated thirteen items on the patients` expectations concerning clinical trials in general practice. Those questionnaires were provided to every enrolled patient of the interventional group as well as the control group of both pilot cluster-randomized trials (*n* = 265) after informed consent by the GP team (online Supplemental file 4). Patients were invited to send their completed questionnaires to the study center (LMU). As a compensation for their expenses, we have offered each survey participant €45.

#### Data collection

We have developed the paper-based questionnaires through iterative procedures and discussion between three researchers (LS, TD, SE and PK). These discussions were informed by specific domains of the TDF. Answers have been provided on a unipolar 4-point Likert scale (from ‘1 = Disagree’ to ‘4 = Strongly agree’; ‘0 = Question unclear’). The collection of the second questionnaires was linked to the time course of the respective intervention, however the perspectives of patients of the intervention group as well as the control group have been evaluated. For this purpose, study participants of pilot study 1 were surveyed via telephone at day 7–14 and day 28, in parallel to scheduled phone contacts that were part of the intervention. Study participants of pilot study 2 received another paper-based questionnaire three months after informed consent, in parallel to a scheduled paper-based evaluation that was part of the intervention (online Supplemental file 5).

#### Data analysis

The quantitative data of the patient questionnaires were analyzed using Microsoft Excel and descriptive, exploratory statistics were generated.

## Results

### The experiences of the GPs

We have invited *n* = 41 GPs and could evaluate *n* = 15 GP interviews finally. The interviewed GPs worked mainly in joint practices (with at least two general practitioners) in a rural area (< 100.000 inhabitants). Prior experience with active clinical research (before both cluster-randomized pilot studies) in their own practice was reported by three of the interviewed GPs, but all of the interviewed GPs had a doctorate in medicine Table 1.

**Table 1:** Characteristics of the interviewed GPs (*n* = 15)

Gender (male)	*n* = 9 (60%)
Practice located in an urban area (> 100.000 inhabitants)	*n* = 4 (27%)
Working in a joint practice	*n* = 14 (93%)
Prior experience with clinical research (before both cluster-randomized pilot studies)	*n* = 3 (20%)
Medical doctorate	*n* = 15 (100%)

Five themes emerged from our analysis of GP interviews: main facilitators for participation in clinical research were networking among involved GPs as well as an active participation in clinical research fostering resource-saving study designs and a well-prepared implementation of upcoming studies. As main barriers, the GPs identified missing awareness of practice-based research networks (PBRNs) in German primary health care as well as a lack of motivation among MAs and patients.

The following presentation of the results is based on the interview guide (see Supplementary File 1). “(…)” means a break in the narrative flow, “[…]” means a shortening of the quote.

1. Networking among involved GPs

The majority of GPs emphasized their aim to improve networking among involved GP teams of BayFoNet. Most interviewees stated that there was no contact to their colleagues so far. Especially personal and regular regional networking with practices in the immediate vicinity was desired.

*“I mean, well, number one might be that, and I don’t know how to make it work with data protection, but let’s see who is close-by and who could get in contact with whom in the first place.” (interview_a06)*

2) Active participation throughout the research process

Committed GPs would like to be actively involved in the development of research questions and implementation of clinical trials in general practice. This includes the active planning of upcoming trials and the evaluation of prior research projects.

*“So far, the finished publication is presented at some point. The question is, whether it is possible to further activate and motivate individual colleagues who really want to get involved.” (interview_c10)*

3) Resource-saving study design and well-prepared implementation

A resource-saving and well prepared structed implementation of clinical studies is important. A major hurdle often described by GPs is the lack of time in everyday practice.

*“Well, the problem in daily routine is always that you really have very little time and that, I think, is often not seen from the outside […] (interview_c08).”*

The effort of the practice team should be reduced as much as possible by organizing the study documents well in advance. For instance, color coding could help to differentiate between documents that are given to patients, sent to the institute or kept in the practice.

*“This is sheet number one, patient, colon. Sheet number one, doctor, colon. So, it was pretty much like that with IMONEDA. But somehow, there were still some things to stumble over or that could be forgotten. So, like, for example, one had yet to prescribe this peak flow meter. And that happened with the first two patients, only afterwards did it occur to us, oh crap, we actually still have to prescribe it. So maybe, for example, just to give an example, one could have included a sample prescription in there. (interview_a05)”*

GPs describe patient recruitment as a very time-consuming process. GPs feel a need for support in recruiting patients. For example, a mobile study assistance to support patient recruitment was suggested.

*“What I regard as very helpful is, for example, when a recruitment team comes to the practice, which will be the case in an upcoming study shortly. (interview_a12)”*

4) Awareness of practice-based research networks (PBRNs)

Almost every interviewed GP mentioned, that PBRNs have to become more popular and better known among GP teams as well as the general public. Particular attention should be paid to the more rural regions. Otherwise, it may be challenging for interested GPs to motivate colleagues as well as patients to participate in clinical research.

*“I personally just found out about this network by chance. And none of my colleagues had ever heard of you or knew what it was at all. It was a bit of conviction to register our practice team.” (interview_a07)*

*“Maybe there is a certain basic interest on the part of the patients. That they know that we are involved, for example now, because that is ultimately what it is all about. We are dependent on their participation. (laughs)” (interview_a07)*

5) Motivation among MAs and patients

Some GPs reported missing motivation among their MAs. Due to staff shortages and a very high workload in daily patient care, not all employees were willing to invest extra time in clinical research.

*I: “Do or are the medical assistants with you then/ Or what could be done so that they might be more interested in such studies?”*

*B: “You tell me. They don’t feel like doing anything.” (interview_a05)*

From the interviewed GPs` point of view, some patients did not recognize the relevance of clinical research and are unwilling to invest time by active participation. Therefore, it is of the utmost importance to address an illness with relevance to the patients.

*“It’s only at the university and everyone is motivated and in the normal setting, the fewest patients are motivated. The patients don’t even take their pills. So why should they participate in a study?” (interview_a01)*

### The experiences of the MAs

A total of 15 MAs were interviewed, who were mainly female (*n* = 14). Most of the interviewed MAs worked in a joint GP practice in a rural area and had a long time of experience in their profession (18 years mean) Table 2.

**Table 2:** Characteristics of the interviewed medical assistants (*n* = 15)

Gender (female)	*n* = 14 (93%)
Practice located in an urban area (> 100.000 inhabitants)	*n* = 2 (13%)
Working in a joint practice	*n* = 6 (40%)
Prior experience with clinical research (before both cluster-randomized pilot studies)	*n* = 3 (20%)
Number of years in the profession	18 years (mean)

Three themes emerged from our analysis: increased professional knowledge and competence and the importance of clinical research for evidence-based patient care as main drivers for participation. Missing awareness and understanding of PBRNs was identified as a main barrier.

1. Increased professional knowledge and competence

The majority of the interviewed MAs appreciated the possibilities the get professionally trained and to gain specific knowledge via regular online courses offered in BayFoNet.

*“Yes, but apart from that, yes, all these events that are always taking place, most of which are online. It’s very practical because you can do it from home. Yes, yes, they do offer a lot, in terms of training and so on.” (interview_m0025)*

A personal interest in research practices was mentioned as well as expected benefits of large networks. From the perspective of some MAs, networks in general might be useful to share competences and collect data to improve patient care as well as the quality of evidence in general practice.

*“Yes, otherwise I find it really interesting, even under the microscope. I think it’s really cool when you see all that, yeah.” (interview_m0023)*

*“Yes. Networks are great. I mean, you have a large area there, right? I think you utilize all the possibilities. You have a large network; one person can consult with another. The more information you can collect from anywhere? The better?” (interview_m0024)*

2) Importance of clinical research for evidence-based patient care

Some MAs assumed that an active participation in clinical research could be a good signal for evidence-based diagnostics and treatment of patients in their practices. Furthermore, active participation in clinical trials could contribute to a good public image of general practice.

*“Yes, for the practice in any case. So, I think it also gives patients a bit of a sense of security when they see that we’re doing this, thar we’re committed to it. And, yes for me personally, I would simply say that it’s helpful for science to take part in something like this, to get involved. Yes.” (interview_m0025)*

3) Awareness of practice-based research networks (PBRNs)

Similarly, to the interviewed GPs, MAs emphasized the need for an increased external presentation and publicity to promote PBRNs among colleagues and patients. They perceived that most patients seemed to be unaware of PBRNs when the study took place in the GP practice.

*B2: “I think that also makes it maybe, I don’t know, more attractive for the patients?”*

*B1: “Yes, exactly.”*

*B2: “Well, if they knew. Often they don’t know either.” (interview_m0029)*

In contrast to the GPs, most MAs were not aware of BayFoNet as an existing PBRN and did not understand that BayFoNet is not just a specific study or a training platform.

*B3: “Okay, very nice, yes. And what added value do you see in the BayFoNet research network?”*

*B4: “I don’t know. I have never heard about something like that.” (interview_m0021)*

### The experiences of the patients

Altogether, *n* = 265 patients could have been recruited in both pilot cluster-randomized trials. Within the pilot cluster-randomized trial 1, *n* = 90 patients in the intervention group and *n* = 67 patients in the control group have been recruited. Pilot cluster-randomized trial 2 enrolled *n* = 62 patients in the intervention group and *n* = 46 patients in the control group.

We have evaluated *n* = 65 questionnaires (response rate: 46%) before the MicUTI intervention was conducted and *n* = 15 questionnaires (response rate: 14%) before the IMONEDA intervention took place.

The surveyed patients of MicUTI felt sufficiently able to participate in the study presented, meaning they had the capability support the clinical trial (item 1–4/ median: 4). In addition, participating patients had the opportunity to participate (item 5 -6/ median: 4). In terms of motivation, the study participants were highly motivated to support the clinical study (item 7–8 and item 10–12/median: 4) However, the motivational domains “emotional regulation” and “goal setting” were slightly affected (item 9 and item 13/ median: 3) Table 3.

**Table 3:** Patient questionnaire before the intervention of pilot-cluster randomized trial 1 (*n* = 65)

Statement	Median
1 I know what the presented study is about and what I can contribute here	4 (1 Missing)
2 I can remember the proper conduct and planned procedure of the study, which I was educated about in my primary care physician’s office	4
3 I can plan my daily routine so that I can participate in the study as discussed with the practice team	4 (1 Missing)
4 I am physically able to participate in the study presented	4
5 My relatives/partner/family support me in participating in the presented study	4
6 I have the necessary material or technical support to participate in the study presented	4 (2 Missings)
7 There are effective incentives to participate in the study	4 (3 Missings)
8 I accept to be randomly assigned to one of the two study groups	4 (6 Missings)
9 As an affected patient, I feel an obligation to other patients to participate in the study presented	3
10 I look forward to actively participating in the study presented	4 (3 Missings)
11 I have clear objectives in participating in the study presented	4 (5 Missings)
12 By participating in the study presented, I would like to help improve medical care for others affected by the disease	4
13 By participating in the study presented, I will be making an important contribution to better patient care	3

Many study participants of IMONEDA asked before the intervention was conducted, agreed that they had the capability to perform the clinical study (item 1–4/ median:4). They strongly agreed, that they had the opportunity to support IMONEDA sufficiently (item 5–6/ median:4). Concerning their motivational domains, these patients had some difficulties recognizing clear goals in their participation. (item 11/ “I have clear objectives in participating in the study presented.”/ median: 2,5) Table 4.

**Table 4:** Patient questionnaire before the intervention of pilot-cluster randomized trial 2 (*n* = 15)

Statement	Median
1. I know what the study presented is about and what I can contribute here	4
2 I can remember the proper conduct and planned procedure of the study that I was educated about in my primary care physician’s office	4
3 I can plan my daily routine so that I can participate in the study as discussed with the practice team	3
4 I am physically able to participate in the study presented	4
5 My relatives/partner/family support me in participating in the presented study	4 (3 Missings)
6 I have the necessary material or technical support to participate in the presented study	4
7 There are effective incentives to participate in the study	3
8 II accept to be randomly assigned to one of the two study groups	4
9 As an affected patient, I feel an obligation to other patients to participate in the study presented	3 (2 Missing)
10 I am looking forward to actively participating in the study presented	3,5 (1 Missing)
11 I have clear objectives in participating in the study presented	2,5
12 By participating in the study presented, I would like to help improve medical care for others affected by the disease	4 (1 Missing)
13 By participating in the study presented, I will be making an important contribution to better patient care	3 (1 Missing)

During the intervention, a total of *n* = 98 patients (response rate: 62%) from the MicUTI trial, and *n* = 11 patients (response rate: 10%) of the IMONEDA trial shared their experiences.

With the experience of the intervention carried out in both pilot cluster-randomized trials, study participants of MicUTI rated their capability, opportunity and motivation as maximally high (item 1–4/ median: 4). Study participants of IMONEDA rated their capability as very high, however they recognized some limitations in terms of opportunities (item 2/ median 3.5) and their own motivation (item 4/ median: 3.5) Table 5.

**Table 5:** Patient questionnaire during the intervention of both pilot-cluster randomized trials

Pilot-cluster randomized trial 1 (MicUTI) *n* = 98	
Statement	Median
1 I know why it is important to continuously participate in the presented study until the end	4 (1 Missing)
2 Participation in the study can be integrated well into my everyday life	4
3 I have the necessary material or technical support to participate continuously in the study presented	4 (2 Missings)
4 I am satisfied with the content and process of the study	4 (1 Missing)
Questionnaire 2: During-intervention	
Pilot-cluster randomized trial 2 (IMONEDA) *n* = 11	
Statement	Median
1 I know why it is important to continuously participate in the presented study until the end	4 (1 Missing)
2 Participation in the study can be integrated well into my everyday life	3,5
3 I have the necessary materials or technical support to continuously participate in the study presented	4 (1 Missing)
4 I am satisfied with the content and process of the study	3,5 (1 Missing)

## Discussion

### Statement of principal findings

Within this mixed-methods evaluation, a total of 15 GPs were interviewed individually. Main facilitators for GPs’ active participation in clinical research were networking as well as the opportunity to participate in the development of upcoming research questions and feasible study designs. However, this does not exclusively mean networking with academic general practice. Rather, it seems important to the GPs to have an exchange with other GPs in the region. As part of PBRNs, project-specific regular roundtables with participating GPs could be useful to improve networking with regard to a specific clinical research question and the respective study design.

A lack of motivation among their staff and their patients was a perceived barrier from the GPs` perspective. The 15 interviewed MAs emphasized their own increase in knowledge and competence as well as the importance of clinical research for improved patient care as main motivators to participate actively in clinical research. With regard to BayFoNet, it was revealed that the organization and objectives of a PBRN has to be more comprehensible for this target group. PBRNs should actively integrate MAs not only in the conduction of a clinical trial, but into the entire research process. Furthermore, many more MAs should be specifically trained in the basics of clinical research so that they are empowered to decide if and in which way they would like to support clinical trials within a PBRN.

The 109 patient questionnaires revealed very good capabilities and opportunities to actively participate in both pilot studies of BayFoNet from the patients` perspective. Prior to the implementations of the interventions, patients of the pilot cluster-randomized trial 2 had experienced some difficulty in defining clear goals for their own participation. In addition to public forums aimed at laypersons, patient representatives and their relatives, it seems important to address eligible study patients in particular. To increase patient motivation, it might be helpful to briefly address the impact of the respective study results on future patient care as part of the informed consent. Furthermore, PBRNs have to become visible and make their goals and working methods known to the general public.

### Strengths and weaknesses of the study

This study provides important insights into experiences of different stakeholders during an active involvement in clinical research within the PBRN BayFoNet. Clear recommendations can be extrapolated, which are transferable to other PBRNs and might be considered for the preparation of future clinical trials in general practice. The study-involvement of GP teams and their patients from different areas, with different levels of experience increases the explanatory power and support generalizability of our findings.

Limitations occurred, as only those GPs, MAs and patients who actively participated in one of both cluster-randomized pilot studies could be evaluated. Only a few patients of the cluster-randomized pilot study 2 (IMONEDA) took part in the survey. Significantly more patients of the pilot study 1 (MicUTI) have been evaluated, as they were interviewed by telephone. The differences of response rates might also reflect the motivational levels of two different patient groups: pilot cluster-randomized trial 1 examined acutely ill (young) women with suspected uncomplicated urinary tract infection, whereas pilot cluster-randomized trial 2 examined chronically ill patients with bronchial asthma. In-depth interviews about patient experience participating in clinical trials would have been a useful adjunct to the survey. Patients who refused to take part in any cluster-randomized pilot study of BayFoNet could not be evaluated.

### Comparison to literature

In contrast to Germany, countries such as Australia, Canada, the Netherlands, the United Kingdom, and the United States have well established PBRNs in general practice [32]. It is worth learning from such networks and deriving recommendations for successful implementation in Germany [33]. Additionally, it is important to integrate expectations and experiences of all stakeholders within the German primary health care system. It is already known, that the scientific connection to other GP teams as well as academia seems to be an important driver for the participation of GP teams in PBRNs [22]. Especially in more rural areas, participation in such networks could be an opportunity to avoid the feeling of isolation [34]. A qualitative study with Dutch GPs showed that group discussions with colleagues can be motivating. As a result, solutions proposed by colleagues can be adopted and practice behavior might be improved [35].

GPs who are members of EGPRN (European General Practice Research Network) also report the need for good networking with both mentors and colleagues. A further step here would be the cooperation of such networks across national borders [36]. Consequently, a major step for the future would be the development of an international research practice network. This could help to ensure that pioneering countries could guide those that have problems with building the infrastructure and funding such networks [36, 37].

Psychological ownership is a fundamental basis to enable GPs and their teams to conduct clinical trials and other studies by continuous education and training and to support patient recruitment, data collection and documentation in ongoing studies. To meet these requirements, BayFoNet has to use a model of generating research questions in partnership with the GP teams, their patients as well as laypeople with the aim of implementing the procedures tested. As lack of involvement in the research process may foster skepticism in GPs towards clinical research [7], BayFoNet aims to create a culture of ownership. This integrative approach is expected to motivate GP teams as well as their patients to participate in clinical research [38, 39].

PBRNs are general practices affiliated with RNCs with the overarching goal to provide and improve patient care. These practices affiliate with one another to investigate questions related to both improving the care they provide and improving their discipline. Consequently, research conducted in PBRNs differs from other multisite research and presents particular planning challenges [40]. Among other aspects, recruiting and selecting study sites as well as the training of GP teams is fundamental. Striking the balance of scientific rigor with practical application of PBRN studies must be addressed throughout these tasks. The provision of well-structured informational fact sheets in advance enables eligible GP teams to make informed decisions about their study participation [41]. Piloting implementation processes, personal training and regular adaptions are helpful to implement acceptable, relevant and feasible clinical trials in general practice [40].

Structured preparation of clinical trials is also helpful to reflect the lack of practice resources. Lack of time in the daily routine of general practitioners and the resulting overload impede successful participation [42]. Thus, research is often less prioritized in the daily practice routine [43]. Consequently, it is important to design studies that require little time from the practice team while offering plenty of support during implementation [44]. It is striking, that all interviewed GPs worked in joint practices. This suggests that those GPs are more likely to have the resources to conduct clinical trials.

Similarly, patient recruitment is a time-consuming process. For general practitioners themselves, recruitment can be challenging for a variety of reasons. For example, there are often very specific inclusion criteria that must be met in order for patients to be eligible for the respective studies. However, this creates uncertainty on the part of GPs and impedes study participation [45]. Support in patient recruitment via mobile study assistants is desirable [46]. Another possible solution could be switching to electronic software designed to facilitate recruitment [47].

An assumed barrier from the GPs` perspective to conduct clinical trials was a lack of motivation among their MAs and patients. From the MAs perspective, patient care and increased professional competences are main motivators for MAs to clinical research. Delegation of a variety of complex tasks might help to improve MA’s job satisfaction and motivation [10]. Therefore, a well-prepared distribution of tasks and structured training could help to increase the job satisfaction of MAs in the short term and improve patient care in the long term. In BayFoNet, we have explicitly invited GPs and their MAs to participate in regular remote professional training in 2023 and 2024. As most of the interviewed MAs had problems recognizing any benefit from active participation in clinical research, they must be actively invited to participate, reached via targeted communication channels and given a clear role in the research process [48, 49].

It is noticeable that some chronically ill patients also had problems identifying clear goals for participation ahead of the intervention. Lack of knowledge about clinical research in general practice as well as PBRNs might be reasons for reduced motivation. Patient and public involvement has gained importance in Germany and is also increasingly implemented in German PBRNs [50]. Strategies and contextual factors that enable optimal engagement of patients vary from low-level engagement (consultative unidirectional feedback) to high-level engagement (co-design or partnership strategies [14]). Therefore, an appropriate environment must also be created for laypersons, patients and eligible study participants. Like MAs, patients or laypersons require appropriate structural support, such as clear descriptions of roles in the research process, adequate training, institutional guidance, regular feedback, appropriate translation services and suitable incentives [51].

### Meaning of the study

Awareness and publicity towards BayFoNet have to be increased and overarching goals of clinical research in general practice have to be communicated tailored to the specific needs of each target group. To identify topics relevant to GP teams and their patients we have introduced different participatory approaches including professional training and dissemination strategies of scientific results within BayFoNet. In the future it will be of the utmost importance to integrate participatory formats and efficient ways of science communication into PBRNs.

## Conclusion

PBRNs are an important element and the foundation for conducting clinical trials in general practice. Awareness of these networks has to be increased and benefits have to be elaborated for all study participants beyond study implementation. Our findings are relevant for the development of primary care research and PBRNs on a national level and may be helpful to elaborate recommendations and indicators for clinical research in general practice on an international level.

## Supplementary Information

Supplementary Material 1. Consolidated criteria for reporting qualitative research (COREQ).

Supplementary Material 2. Interview guide for general practitioners.

Supplementary Material 3. Interview guide for medical assistants.

Supplementary Material 4. Questionnaire for patients (before the intervention).

Supplementary Material 5. Questionnaire for patients (during the intervention).

## Data Availability

Data and materials might be obtained from the authors upon reasonable request.

## Abbreviations

BayFoNet: Bavarian Research Practice Network
BMBF: German federal ministry of education and research
CFIR: Consolidated Framework for Implementation Research
GP: General practitioner
MA: Medical assistant
PBRN: Practice-based research network
TDF: Theoretical domains framework
RNC: Regional network centers
