# Supplementary material for: Experiences of general practice teams and their patients with clinical research—a mixed-methods process evaluation of the Bavarian Research Practice Network (BayFoNet)
Source: BMC Prim Care. 2025 Feb 28;26:59. doi: 10.1186/s12875-025-02744-x (PMC11869661; doi:10.1186/s12875-025-02744-x)
Supplement: Supplementary file 1 — Supplementary Material 1. Consolidated criteria for reporting qualitative research (COREQ). [file 12875_2025_2744_MOESM1_ESM.docx]

<online supplemental file 1: Interview guide for the general practitioners>

| 1. What added value does participation in BayFoNet have for your practice? |
| --- |
| 1. What added value does conducting clinical trials have for you and your practice? |
| 1. How can BayFoNet provide practices with even better support in the implementation of clinical trials? |
| 1. What characteristics must clinical trials have to make them attractive and feasible for patients? |
| 1. What changes have you experienced in practice as a result of conducting a clinical trial? |
| 1. What barriers have you identified so far in conducting clinical trials? |
| 1. How well have you been prepared for participating in a clinical trial as part of BayFoNet? |
| 1. Is there an exchange with colleagues from other practices regarding your research? |
| 1. Can you imagine integrating clinical studies into your everyday practice in the long term? |
| 1. Is there anything you would like to say that has not yet been mentioned? |
