# Supplementary material for: Experiences of general practice teams and their patients with clinical research—a mixed-methods process evaluation of the Bavarian Research Practice Network (BayFoNet)
Source: BMC Prim Care. 2025 Feb 28;26:59. doi: 10.1186/s12875-025-02744-x (PMC11869661; doi:10.1186/s12875-025-02744-x)
Supplement: Supplementary file 2 — Supplementary Material 2. Interview guide for general practitioners. [file 12875_2025_2744_MOESM2_ESM.docx]

<online supplemental file: interview guide for medical assistants>

| 1. What added value does conducting clinical trials have for you? |
| --- |
| 1. What added value does participation in BayFoNet have for you? |
