# Supplementary material for: Experiences of general practice teams and their patients with clinical research—a mixed-methods process evaluation of the Bavarian Research Practice Network (BayFoNet)
Source: BMC Prim Care. 2025 Feb 28;26:59. doi: 10.1186/s12875-025-02744-x (PMC11869661; doi:10.1186/s12875-025-02744-x)
Supplement: Supplementary file 4 — Supplementary Material 4. Questionnaire for patients (before the intervention). [file 12875_2025_2744_MOESM4_ESM.pdf]

Patient questionnaire

„Process evaluation for the implementation of clinical studies in the Bavarian Practice-Based Research Network (BayFoNet)- part 1“

Please mark the appropriate box clearly.

Please express your agreement with the following statements on the following scale:

**1**	**2**	**3**	**4**	**0**
Disagree	Strongly disagree	Agree somewhat	Strongly agree	Question unclear

Statement					
1. I know what the study presented is about and what I can contribute here.	☐ 1	☐ 2	☐ 3	☐ 4	☐ 0
2. I can remember the proper conduct and planned procedure of the study that I was educated about in my primary care physician's office.	☐ 1	☐ 2	☐ 3	☐ 4	☐ 0
3. I can plan my daily routine so that I can participate in the study as discussed with the practice team	☐ 1	☐ 2	☐ 3	☐ 4	☐ 0
4. I am physically able to participate in the study presented.	☐ 1	☐ 2	☐ 3	☐ 4	☐ 0
5. My relatives/partner/family support me in participating in the presented study.	☐ 1	☐ 2	☐ 3	☐ 4	☐ 0
6. I have the necessary material or technical support to participate in the presented study.	☐ 1	☐ 2	☐ 3	☐ 4	☐ 0

Please turn the sheet→

Please express your agreement with the following statements on the following scale:

1	2	3	4	0
Disagree	Strongly disagree	Agree somewhat	Strongly agree	Question unclear

Statement	1	2	3	4	0
7. There are effective incentives to participate in the study.	☐	☐	☐	☐	☐
8. I accept to be randomly assigned to one of the two study groups.	☐	☐	☐	☐	☐
9. As an affected patient, I feel an obligation to other patients to participate in the study presented.	☐	☐	☐	☐	☐
10. I am looking forward to actively participating in the study presented.	☐	☐	☐	☐	☐
11. I have clear objectives in participating in the study presented.	☐	☐	☐	☐	☐
12. By participating in the study presented, I would like to help improve medical care for others affected by the disease.	☐	☐	☐	☐	☐
13. By participating in the study presented, I will be making an important contribution to better patient care.	☐	☐	☐	☐	☐

Thank you very much,

for taking the time to participate in this study today!

Your commitment is important to us. By taking part, you are making a valuable contribution to research into medical care in Germany.
