# Supplementary material for: Experiences of general practice teams and their patients with clinical research—a mixed-methods process evaluation of the Bavarian Research Practice Network (BayFoNet)
Source: BMC Prim Care. 2025 Feb 28;26:59. doi: 10.1186/s12875-025-02744-x (PMC11869661; doi:10.1186/s12875-025-02744-x)
Supplement: Supplementary file 5 — Supplementary Material 5. Questionnaire for patients (during the intervention). [file 12875_2025_2744_MOESM5_ESM.pdf]

Patient questionnaire

„Process evaluation for the implementation of clinical studies in the Bavarian Practice-Based Research Network (BayFoNet)- part 2“

Please mark the appropriate box clearly.

Please express your agreement with the following statements on the following scale:

**1**	**2**	**3**	**4**	**0**
Disagree	Strongly disagree	Agree somewhat	Strongly agree	Question unclear

Statement					
1. I know why it is important to continuously participate in the presented study until the end.	☐ 1	☐ 2	☐ 3	☐ 4	☐ 0
2. Participation in the study can be integrated well into my everyday life.	☐ 1	☐ 2	☐ 3	☐ 4	☐ 0
3. I have the necessary materials or technical support to continuously participate in the study presented.	☐ 1	☐ 2	☐ 3	☐ 4	☐ 0
4. I am satisfied with the content and process of the study.	☐ 1	☐ 2	☐ 3	☐ 4	☐ 0

Thank you very much,

for taking the time to participate in this study today!

Your commitment is important to us. By taking part, you are making a valuable contribution to research into medical care in Germany.
